# Molecular diagnosis of arboviruses (dengue, Chikungunya, yellow fever, Rift Valley fever, Mayaro, West Nile, and Zika viruses) in non-malaria acute febrile illnesses in Kenya’s Garissa County, July 2023, and Mombasa County, July 2024

**DOI:** 10.1186/s12879-025-11719-3

**Published:** 2026-01-09

**Authors:** Abdi Roba, John Kiiru, Elvis Kirui, Paul Langat, Marygorett Mbeneka, Mellap Wanyonyi, Judith Nasengo, Hakim I. Lagu, Emmanuel Achol, Muna Affara, Florian Gehre, Hussein Hassan, Evans Odiambo, Fatuma Dugay, Aden Hussein, Frankline Okemwa, Salma Swaleh, Suleiman Thani, Rose Gichana, Isaiah Karissa, Millicent Ndia

**Affiliations:** 1 Ministry of Health, Nairobi, Kenya; 2 Division of National Laboratory Services, Nairobi, Kenya; 3 Health Department, East African Community (EAC) Secretariat, Arusha, Tanzania; 4 Infectious Disease Epidemiology Department, Bernhard-Nocht-Institute for Tropical Medicine, Hamburg, Germany; 5 Garissa Teaching and Referral Hospital, Garissa, Kenya; 6 Public Health Department, Mombasa, Kenya; 7 Mombasa Department of Health services, Mombasa, Kenya; 8 Public Health Department, Mvita Sub County Hospital, Mombasa, Kenya

**Keywords:** Mobile laboratory, Arbovirus, East africa, Kenya, Viral haemorrhagic fever

## Abstract

**Background:**

Viral haemorrhagic fever outbreaks can have a devastating impact on local populations. In Mombasa County, and also remote Kenyan areas, such as Garissa County, where health centres have limited diagnostic capacity, the causes of non-malaria, febrile illnesses are often unknown. In particular mosquito-borne, arboviral infections, which can lead to severe disease manifestations, are difficult to detect without molecular diagnostics. The present study aimed to establish the prevalence of selected arboviral agents in blood samples from patients presenting with non-malarial febrile illness at health facilities in Garissa and Mombasa County.

**Methods:**

Following the long rainy seasons and during peak-transmission season for mosquito-borne diseases, the National Public Health Laboratory, Ministry of Health, Kenya, deployed a mobile laboratory with molecular testing capacity to the remote Garissa County Referral Hospital (GCRH) in July 2023 and to Mvita Sub-County Hospital in Mombasa County in July 2024. With the help of this mobile laboratory, a prospective arboviral surveillance mission was conducted for the first time in Garissa County in north eastern Kenya bordering Somalia, as well as in the coastal region of Kenya’s Mombasa County. These, and neighbouring counties reported arboviral infections before.

**Results:**

A total of 326 febrile patients from Garissa, for which malaria microscopy was negative, received differential diagnosis for risk group 3 arboviruses using the Altona Diagnostics Real Star RT-PCR kits for dengue, Chikungunya, Rift Valley, yellow fever viruses. We found four dengue virus PCR-positive patients and therefore 1.23% (CI95%: 0.36%-3.23%, modified Wald method) of non-malaria febrile illnesses were due to this virus. In Mombasa County, 289 non-malaria, febrile patients cases were screened with the VIASURE Tropical Panel I Real Time PCR Detection Kit for dengue, Chikungunya, yellow fever, Zika, Mayaro, West Nile viruses. We found 26 PCR-positive patients for dengue virus or a dengue prevalence of 9.00% (CI95%: 6.17%-12.90%). We did not detect any of the other mosquito-borne arboviruses, however we cannot exclude their role in future outbreaks in the region following rainy and mosquito seasons.

**Conclusions:**

Although molecular diagnostics exist at the central NPHL, our findings show that in peripheral health centres in both counties, scaling-up of arboviral diagnostics in general, and dengue diagnostic capacity (rapid tests, PCR, serology) in particular, for patients presenting with febrile illness is urgently needed. As one of the novel dengue vaccine regulations recommend that only previously infected patients receive the vaccine in order to prevent severe forms of the disease, increased surveillance, serotyping of isolates and host serology is essential as well. The mobile laboratory demonstrated its added value to provide diagnostic services to remote cross-border areas.

**Supplementary Information:**

The online version contains supplementary material available at 10.1186/s12879-025-11719-3.

## Introduction

Sub-Saharan Africa (sSA) is home to many non-malarial acute febrile illnesses, but viral haemorrhagic fever viruses and arboviruses have emerged among significant causes. Both present important veterinary and public health threats, where they cause a number of syndromes including viral haemorrhagic fever, encephalitis and arthritis [1–4]. The WHO technical working group on arboviruses expects further spread of arboviruses due to changing ecologic, economic and social factors [5]. A recent meta-analysis showed eight arboviruses are well established in sSA, seven of which were found in Kenya already [6]. For instance, dengue fever was first discovered in Kenya in Mandera in September and October 2011 [7], and Mombasa in 2013 [8]. The first yellow fever outbreak in Kenya was reported in 1992. More outbreaks were recorded in the western parts of the country (1993, 1995, 2011) particularly in the Rift Valley province of Kenya [9]. In March 2022, yellow fever re-emerged in Kenya, affecting 11 wards in Isiolo County [9]. In the same county, 18.3% of abattoir workers were found to have antibodies against Rift Valley fever virus [10]. The largest Chikungunya outbreak happened in Lamu in 2004 [11] and was again detected in Mombasa from late 2017 to early 2018 [12] and Mandera in May 2016 [13].

There is an overarching challenge in diagnosis and surveillance of these pathogens, owing to the generic nature of symptoms and the lack of access to specialised diagnostics. In particular, risk group 3 and 4 arboviruses require mechanisms to safely receive and inactivate these viruses prior to PCR diagnosis. As seen during the recent Ebola virus outbreaks in East Africa [14], the needed infrastructure and laboratory capacity are often not readily available in remote, cross border areas where viral outbreaks occur. With limited epidemiologic arboviral surveillance data, identifying causative agents of non-malarial acute febrile illness in Kenya is important for improving patient management and public health interventions.

In the present publication, we describe the findings of two consecutive arboviral surveillance studies in two Kenyan counties. Garissa County in north eastern Kenya not only borders Somalia and is home to a significant refugee population but also borders Isiolo and Lamu counties where previous arboviral outbreaks were detected. Mombasa County was previously described as a region with various arboviral outbreaks [8, 12]. Besides generating arboviral surveillance data, we demonstrate that, instead of shipping samples to the central NPHL in Nairobi, a mobile laboratory can significantly reduce the diagnostic sample turn-around-time and accelerate patient diagnosis. Moreover, we showed for the first time that it is a powerful solution to provide diagnostic services for hard-to-reach Kenyan/Somalian cross border regions, which are often underserved.

## Methods

### Aim of Study

The main aim of this study was to investigate the prevalence of selected arboviral agents in blood samples from patients presenting with non-malarial febrile illness at health facilities in Garissa County and Mombasa County. In order to achieve this, and following the rainy seasons (March-June) and subsequent mosquito blooms, the Kenyan National Public Health Laboratory (NPHL) under the Ministry of Health, deployed one NPHL mobile laboratory in July 2023 to the Garissa County Referral Hospital (GCRH) and in July 2024 to the Mvita Sub-County Hospital (MSCH) in Mombasa County. With provision of the needed laboratory capacity to the region we conducted arboviral surveillance amongst non-malaria febrile patients in north eastern and coastal Kenya.

### Surveillance area in Garissa County and Mombasa County

Garissa County is located in the former north eastern province of Kenya and covers an area of 44.176 km^2^. Garissa County is located approximately 350 km and 450 km from Nairobi or Mombasa, respectively, and due to security issues and inaccessibility, parts of Garissa can only be reached by military convoy and with offroad-capable vehicles. It borders Wajir County to the north, Tana River County to the west, Isiolo County to the north west, Lamu County to the south and the Federal Republic of Somalia to the east. The county has a population of 841.353 persons [15], with a refugee population of 226.702 persons. It is home to three livelihood zones namely: (i) pastoral (camels, goat, sheep and cattle), (ii) agro-pastoral, and (iii) formal employment [16]. Garissa County has eleven sub-counties aggregated into six constituencies which include: Fafi, Garissa Township, Ijara, Lagdera, Balambala and Dadaab. Further, Garissa County is served by the Garissa County Referral Hospital (GCRH), sub-county hospitals, 56 dispensaries, 21 health centres and 123 private clinics and nursing homes. Due to its proximity to Somalia, the distant Garissa County with its large refugee population is facing serious security issues and is exposed to cross-border epidemics with only minimal, specialised laboratory capacity.

Mombasa County with Mombasa city as its capital, is Kenya’s second most economically developed county after Nairobi city. It is the smallest county in Kenya, covering an area of 229.7 km^2^ excluding 65 km^2^ of water mass. The county is situated in the south eastern part of the former Coast Province, bordering Kilifi County to the north, Kwale County to the south west and the Indian Ocean to the east. It has 360 health facilities spread across the six sub-counties (Mvita, Changamwe, Kisauni, Jomvu, Nyali, and Likoni), of which 15 are faith-based facilities, 60 belong to the Ministry of Health, 273 private facilities and 12 under NGO’s operations.

### Mobile Laboratory Structure

**Fig. 1:**
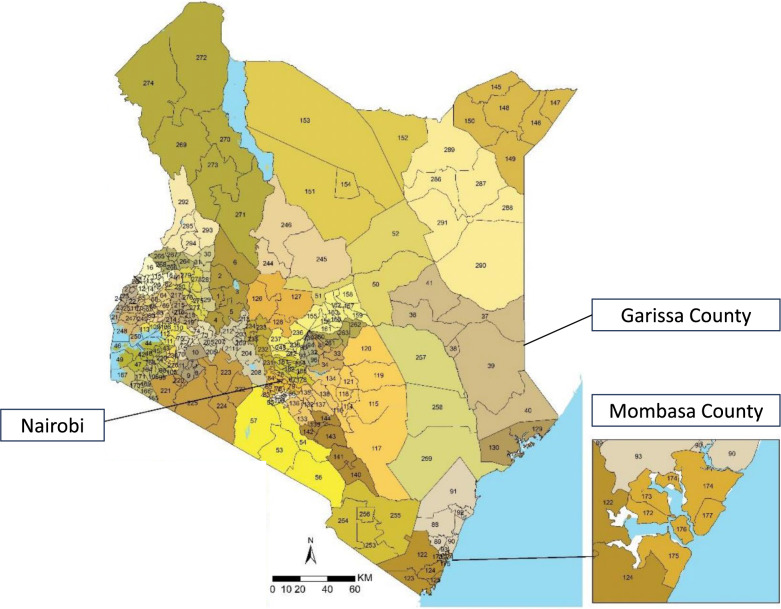
Sub-counties in Kenya and mobile laboratory deployment sites. Garissa County consists of Balambala (36), Dadaab (37), Garissa township, where mobile laboratory was located (38), Fafi (39), Ijara (40) and Lagdera (41). Mombasa County consists of Changamwe (172), Jomvu (173), Kisauni (174), Likoni (175), Mvita where mobile laboratory was located (176) and Nyali (177) (map was adapted from [18])

The Kenyan NPHL mobile laboratory is part of the wider East African Community (EAC) mobile laboratory network [17]. The laboratory can safely receive, inactivate and diagnose risk group 3 and 4 pathogens. For further information on mobile laboratory setup and diagnostic workflows please refer to [17]. During respective study periods, the NPHL mobile laboratory was stationed at Garissa County Referral Hospital (GCRH) in Garissa County and at Mvita Sub-County Hospital (MSCH) in Mombasa County (Fig. 1).

The mobile laboratory team comprised of four laboratory experts from the centralised NPHL, Nairobi, who were previously intensively trained under the EAC mobile laboratory project [17]. In both deployment locations the laboratory team followed an identical approach to setup the laboratory in respective hospitals and integrate it into sample referral and reporting systems of the counties (see supplemental Fig. 1). In brief, after consultation with the Director of Public Health and the County Medical Laboratory Coordinator (CMLC) an adequate room with sufficient lighting, electrical sockets, working benches and hand washing sink was identified. All mobile laboratory work stations were set-up within the room, considering pre- and post-PCR areas. In both hospitals functional incinerators were available and used for waste management of the mobile laboratory. All mobile laboratory equipment were tested by successfully diagnosing one known PCR positive and one known PCR negative mock dengue blood sample, provided by the NPHL Nairobi. In both locations, the NPHL team trained an additional two laboratory officers on sample reception, use of glove box, biosafety, RNA extraction, quality control and PCR. Before operations commenced, the mobile laboratory team lead and the CMLC visited near-by health facilities not only to observe patient flow and screening activities, but also to create awareness to ensure referral of all malaria negative samples from patients with febrile illness to the mobile laboratory. In Garissa, far flanked sub-county facilities were not visited because of distance and security issues, however virtual briefings were conducted.

### Patient diagnosis

Blood samples in EDTA tubes from patients that met a predefined case definition (fever > 37.5 °C with two or more symptoms such as myalgia, arthralgia and headache) and who tested negative for malaria by microscopy, were referred to the mobile laboratory for further molecular confirmation. Sub-county hospital laboratories packaged and shipped specimens on the same day in cold chain (in insulated transport boxes with ice-packs) to the NPHL mobile laboratory stationed at GCRH and MSCH, respectively. Although no temperature loggers were used, cold arrival of samples at the mobile laboratory was confirmed during sample reception. Samples were inactivated in a glovebox, using Qiagen AVL buffer and ethanol, and viral RNA was extracted from inactivated samples using the Qiagen Viral RNA Extraction kit. PCR testing was performed on the BioRad CFX96 real-time thermocycler. In Garissa County we tested for dengue, Chikungunya, Rift Valley and yellow fever viruses using four single-plex Real Star RT-PCR kits (Altona Diagnostics, Hamburg, Germany). In Mombasa, molecular diagnosis was done for dengue, Chikungunya, yellow fever, Zika, West Nile and Mayaro viruses using the Viasure Tropical Panel 1 RT-PCR detection Kit (Certest Biotec S.L., Zaragoza, Spain). As these are commercial kits, the exact primer and probe sequences are unknown and all kits were used as per manufacturer’s instructions.

## Results

### Demographics of the study population

For both surveys, samples from febrile patients which fulfilled the case definition and were all negative for malaria (based on microscopy) were collected during peak mosquito season in peripheral health centres and referred to the mobile laboratory. For a description of gender and age distribution at both study sites see Tables 1 and 2. The higher amount of “unknown” or missing data (for gender 3%, age 21%) in the Garissa study population, is the reflection of the remote location of the surveillance area.

**Table 1:** Demographic characteristics of Non-Malaria febrile patients in Garissa (July 2023) and Mombasa (July 2024)

Gender	Garissa County	Mombasa County
Female	170 (52%)	184 (64%)
Male	146 (45%)	105 (36%)
Missing	10 (3%)	-
Total	326	289

**Table 2:** The age distribution of Non-Malaria febrile patients in Garissa (July 2023) and Mombasa (July 2024)

Age Group (Years)	Garissa study population	Mombasa study population
0–10	15 (5%)	40 (14%)
11–20	38 (12%)	55 (19%)
21–30	39 (12%)	77 (27%)
31–40	77 (24%)	47 (16%)
41–50	67 (20%)	43 (15%)
> 51	20 (6%)	27 (9%)
Unknown	70 (21%)	-
Total	326	289

### Dengue fever prevalence estimates

In Garissa County, between 12th to 24th July 2023, the mobile laboratory received a total of 326 samples from 14/21 health centers of all constituencies (Balambala, Dadaab, Fafi, Garissa, Ijara, Lagdera), including areas bordering Somalia. None of the screened samples were PCR-positive for Chikungunya virus, yellow fever virus and Rift Valley Fever virus. This suggests low or no circulation of these viruses at time of sampling. We, however, found two female adults (2/170) and two male adults (2/146) that tested positive for dengue virus. Three of the cases were identified in Dadaab sub-county, with a large refugee population and bordering Somalia, and the fourth one was identified from Garissa County Regional Hospital. For three cases no additional demographic data was available, whereas the one female case from Dadaab County was 38 years old. Therefore, around 1.23% (CI95%: 0.36%−3.23%, modified Wald method) of non-malaria febrile illnesses were due to dengue fever (Table 3). The average testing turn-around-time (TAT) in the mobile laboratory, from sample reception to results release, was six hours. Although the TAT is comparable to the central NPHL in Nairobi, we avoided the time-consuming and expensive sample transport from Garissa to Nairobi, which can take days.

**Table 3:** Details of samples tested for the different arboviruses at GCRH in Garissa County and sub-county population size

Constituency/Sub-county (population size)	Facility	No. of samples	No. RT-PCR positive samples			
			Dengue	Chikungunya	Yellow fever	Rift Valley
Balambala (pop.: 32.257)		9	0	0	0	0
Dadaab (pop.: 185.252)	Dadaab	17	3	0	0	0
Fafi (pop.: 134.040)	Hagadera	0	0	0	0	0
	Bura	5	0	0	0	0
Garissa (pop.: 163.914)	Iftin	8	0	0	0	0
	GCRH	153	1	0	0	0
	Madina	15	0	0	0	0
	GK Prison	39	0	0	0	0
	Bula Mzuri	0	0	0	0	0
	Alliance Hospital	12	0	0	0	0
Ijara (pop.: 275.575)	Holugho	11	0	0	0	0
	Ijara	7	0	0	0	0
Lagdera (pop.: 50.315)	Modogashe	44	0	0	0	0
	Shantabaq	6	0	0	0	0
**Total**		**326**	**4**	**0**	**0**	**0**

In Mombasa, the survey was conducted a year later and over a period of two weeks between 8th – 24th, July 2024. A total of 291 samples, which had tested negative for malaria were referred from 21 health facilities within Mombasa County to Mvita Health Centre where the NPHL mobile laboratory was stationed. Out of 291 collected samples, two haemolysed samples were rejected while 289 samples were received and tested for dengue, Chikungunya, yellow fever, Zika, West Nile and Mayaro viruses. Of 289 samples, 26 samples or 9.00% (CI95%: 6.17%−12.90%), modified Wald method) tested positive for dengue virus. No other virus was detected with the multiplex assay (Table 4). In Mombasa County, the average age of dengue PCR positive cases was 28.1 years, which was similar to 28.4 years of dengue PCR negative patients. We also did not find any association between gender and a positive dengue PCR result (Fisher’s exact test, *p* = 0.5374).

**Table 4:** Details of the samples tested at MSCH for the different arboviruses in Mombasa County

Facility	No. of samples	No. of RT-PCR positive samples					
		Dengue	Chikungunya	Yellow fever	Zika	West Nile	Mayaro
Mikindani	38	3	0	0	0	0	0
Mvita	11	1	0	0	0	0	0
Ganjoni	4	1	0	0	0	0	0
Statehouse	18	1	0	0	0	0	0
Magongo	14	2	0	0	0	0	0
Jomvu	13	1	0	0	0	0	0
Chaani	23	3	0	0	0	0	0
Borstal	14	2	0	0	0	0	0
Miritini	29	0	0	0	0	0	0
Mwembe Tayari	31	3	0	0	0	0	0
Shimo Annex	15	1	0	0	0	0	0
Mlaleo	4	0	0	0	0	0	0
Kisauni	7	0	0	0	0	0	0
Port Reitz	17	4	0	0	0	0	0
Shimo Main	13	1	0	0	0	0	0
Majengo	13	2	0	0	0	0	0
Bamburi	11	0	0	0	0	0	0
Kaderbouy	2	0	0	0	0	0	0
King’orani	4	0	0	0	0	0	0
Tononoka	3	1	0	0	0	0	0
Vikobani	5	0	0	0	0	0	0
**Total**	**289**	**26**	**0**	**0**	**0**	**0**	**0**

## Discussion

The present study determined the prevalence of arboviral febrile illnesses of malaria negative patients at Garissa County and Mombasa County health facilities in Kenya, utilizing an NPHL mobile laboratory that can provide required molecular diagnostic capacity to remote areas in Kenya. We found 30 dengue confirmed cases, with acute infection, which, under normal circumstances might have been missed. Although we did not find Chikungunya, Rift Valley fever, West Nile, Zika, or yellow fever viruses in our specific setting and time, possibly due to the short sampling period and low viral interepidemic circulation, we cannot exclude the occurrence of these infections in future. With exception of Mayaro virus, which has never been detected in Africa, other arboviruses were previously described in surveilled or neighbouring counties and should always be included in the primary screening panel during initial stages of an outbreak, in addition to dengue virus. This is important for early outbreak containment, vector control or clinical patient triage. Due to possible complications of acute dengue, Chikungunya, yellow fever and Rift Valley fever infections, it is critical to distinguish between them. Dengue fever can progress to a severe form, characterized by haemorrhages and shock [1]; Rift Valley fever typically presents with a flu-like illness in humans, though a subset of cases may progress to severe manifestations such as haemorrhagic fever, encephalitis, or ocular disease. In livestock, the disease is primarily characterized by high rates of abortion [2]; Chikungunya frequently causes debilitating polyarthritis [3], while yellow fever virus causes liver damage leading to jaundice and haemorrhage [4].

Surprisingly, the identified dengue patients in Garissa, were infected following a long drought in north eastern Kenya and Somalia, with lack of several general rainy and mosquito seasons in the region in the years up to the long rains in March-June, 2023. Following several normal rainy seasons with the consequent, recurrent mosquito bloom, it is highly possible for future dengue fever case numbers to be substantially higher than the 1.23% of non-malaria febrile patients recorded in this study. In previous mosquito surveillance studies, Garissa County was shown to have one of the highest mosquito abundances in the country [19]. In November 2023, after our study, and most likely due to the global El Niño phenomenon, Garissa County was affected by severe floods, which may have resulted in an increase of mosquito-borne infections. In the year following the Garissa survey, and after normal rainy seasons in 2024, we observed a notable increase in dengue cases in Mombasa County, with a prevalence of approximately 9% among non-malaria febrile cases.

Currently there is no national protocol for a diagnostic dengue algorithm in Kenya. Therefore, our first key recommendation based on our findings, is for health facilities to urgently consider dengue virus testing as a differential diagnosis for malaria during rainy season, and to encourage laboratory facilities to conduct PCR-tests (if available) or utilize antigen-based dengue fever rapid diagnostic tests (RDT) for patients during the acute phase of the infection (up to seven days after start of fever), followed by IgM RDTs for patients that delayed presentation to the clinic for more than seven days after onset of symptoms. Three dengue patients were identified in Kenya’s Dadaab County. As Dadaab County borders Somalia, it is advisable that Somalia also increases its dengue fever surveillance in respective cross border regions. This could be done via mobile laboratories, or within existing health facilities. From 2025 onwards, Somalia will be integrated into the EAC mobile laboratory project, and will potentially be equipped with a mobile laboratory for cross-border disease surveillance in future [20].

Secondly, besides rapid patient diagnosis for improved patient treatment, further molecular serotyping of viral isolates by PCR is needed for surveillance. Knowing current (PCR) and previous exposure (ELISA) of a patient to the four dengue virus serotypes 1–4 is crucial in light of the increased roll-out of novel dengue vaccines. This is because during the primary infection with one serotype, the human host’s humoral immune response develops serotype-specific antibodies, which ultimately lead to virus neutralization [21]. Upon secondary infection with one of the other three serotypes, however, these existing antibodies have a lower avidity to the new serotype and aid in viral uptake into host cells, viral multiplication and high viral loads rather than neutralization [21]. This antibody-dependent enhancement of infection (ADE) can result in clinically severe forms of dengue [21], and can especially be problematic for children. In the US, therefore, where the Dengvaxia vaccine obtained approval in February 2022, it is recommended that all children between 9 and 16 years receive the vaccine, but only if they have been previously infected with dengue virus [22]. A previous infection has to be laboratory confirmed either by PCR or IgM serology [22]. In this patient group, the vaccine can efficiently prevent severe forms of dengue fever [22]. An alternative vaccine Q-Denga (TAK-003), in which, according to WHO [23], no prior serological screening is needed, could be an alternative in Kenya, which does not have a national dengue vaccine recommendation yet. Similar public health interventions could be relevant for other East African countries such as coastal Tanzania, which already experienced outbreaks of serotypes DENV-1 and DENV-3 between 2017 and 2019 [24], or Somalia. Therefore, systematic dengue surveillance could inform targeted vaccine rollout strategies at a population level.

The NPHL mobile laboratory demonstrated that it is not only fully operational in Kenya, but that it can deliver urgently needed arboviral diagnostic services to affected areas. Together with a team of well-trained staff from the NPHL in Nairobi, it was successfully transported overland to its destinations, set-up at two different health centers and fully integrated in regional sample-referral systems. The laboratory can provide (i) primary dengue PCR diagnostic and PCR-based serotyping capacity during dengue fever outbreaks, (ii) and ELISA-based IgG/IgM serology for large scale, population-based dengue virus exposure studies during or even outside of outbreak season. Results of the latter can identify previously exposed individuals which would qualify to receive the dengue vaccine. As many arboviral infections are highly seasonal, a temporarily deployed mobile laboratory is an ideal solution for remote African areas, in which permanent year-round diagnostic PCR capacity in stationary laboratories cannot easily be achieved. With its deployment to Garissa County, we demonstrated that the mobile laboratory is a particular good solution for cross-border areas with hard-to-reach populations, and should be integrating in national surveillance or outbreak response activities in future. Within the abovementioned EAC mobile laboratory network, for instance, the NPHL/Kenya received two mobile laboratories, which were utilized in two Kenyan border regions during the COVID-19 pandemic for several years and Mpox screening in 2025 [25], indicating the usefulness and sustainability of such initiatives.

Our study has several limitations:

1. we were not able to physically visit all health centres in Garissa County due to security reasons and sample referral might have been negatively biased. To mitigate this and in addition to physical visits, the CMLCs of both counties had weekly virtual meetings with all health centers prior to arrival of the mobile laboratory. During these briefings health centers were sensitized about the mobile laboratory itself, its diagnostic portfolio and sample referral. Overall, in Garissa 14/21 health centers from all sub-counties, including those from far flanked regions, referred samples to the laboratory, indicating minimal bias.
2. malaria microscopy has limitations for low parasitaemia, mixed species infection and suboptimal slide preparation. The NPHL Kenya operates an external quality assurance system in which peripheral health centres in counties receive blinded microscopy slides for correct reading. However, we cannot exclude that some of the referred samples in which arboviral diagnosis was requested but no virus was detected, were still attributable to underlying malaria infections missed by microscopy.
3. the use of two different PCR kits in the studies was due to varying kit availability at respective timepoints.
4. as we based our dengue diagnosis on RNA detection, there is the possibility that PCR-negative yet IgM positive dengue cases were missed.
5. we did not further serotype identified dengue samples, as the primary goal was an initial screening of arboviruses in the field.
6. we did not find other arboviruses, possibly due to several reasons: (i) the short, two weeks deployment period and screening window and a conceivable low interepidemic circulation of these viruses, (ii) the unknown, and potentially low sensitivities of used commercial diagnostics kits.

For future, possibly longer, mobile laboratory activities, it is of essence to further validate sensitivity of diagnostic kits in East Africa and provide dengue serotyping PCR kits for profiling of dengue cases. It is also advisable to collect more demographic data, such as occupation, refugee status, travel history and onset of symptoms to conduct a more in-depth risk factor analysis, which was not done in our set-up.

Nevertheless, our findings are a first step to inform Kenyan (and possibly Somalian) public health practitioners on the importance of differential diagnosis during suspected febrile illness outbreaks leading to accurate and timely clinical decision making, better intervention response, and vaccination campaigns. Emergence of arboviruses in different parts of Kenya requires that surveillance be increased and that genomic epidemiology be applied to track changes in the viruses genetic makeup, to understand the virus’ genetic population structures, and to study transmission patterns.

## Supplementary Information

Supplementary Material 1.

## Data Availability

All available data is displayed in the manuscript.
