# Supplementary material for: Molecular diagnosis of arboviruses (dengue, Chikungunya, yellow fever, Rift Valley fever, Mayaro, West Nile, and Zika viruses) in non-malaria acute febrile illnesses in Kenya’s Garissa County, July 2023, and Mombasa County, July 2024
Source: BMC Infect Dis. 2026 Jan 9;26:27. doi: 10.1186/s12879-025-11719-3 (PMC12784526; doi:10.1186/s12879-025-11719-3)
Supplement: Supplementary file 1 — Supplementary Material 1. [file 12879_2025_11719_MOESM1_ESM.pptx]

## Slide 1
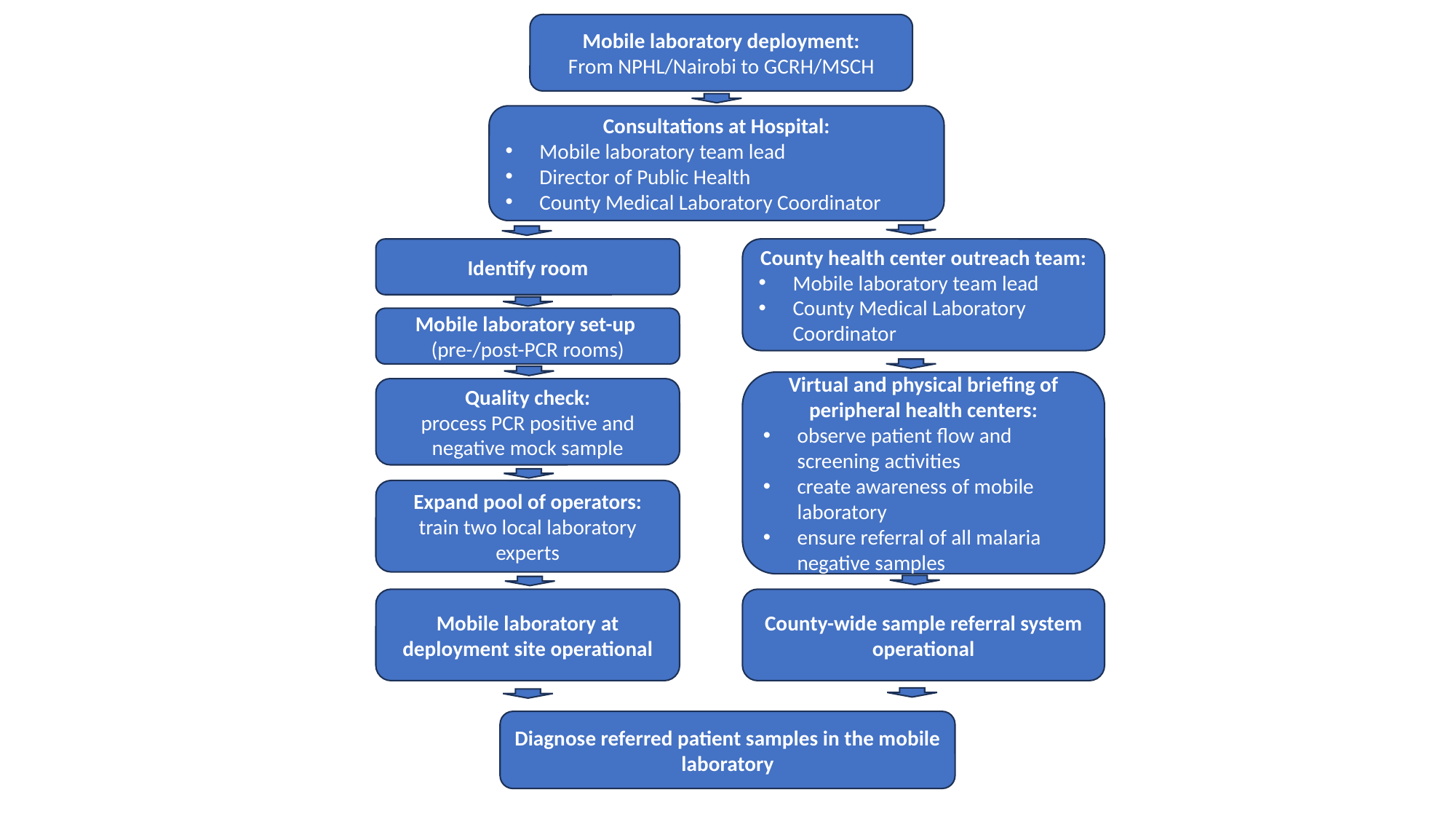

Mobile laboratory deployment:
From NPHL/Nairobi to GCRH/MSCH
Consultations at Hospital:
Mobile laboratory team lead
Director of Public Health
County Medical Laboratory Coordinator
Identify room
County health center outreach team:
Mobile laboratory team lead
County Medical Laboratory Coordinator
Mobile laboratory set-up
(pre-/post-PCR rooms)
Virtual and physical briefing of peripheral health centers:
observe patient flow and screening activities
create awareness of mobile laboratory
ensure referral of all malaria negative samples
Quality check:
process PCR positive and negative mock sample
Expand pool of operators:
train two local laboratory experts
Mobile laboratory at deployment site operational
County-wide sample referral system operational
Diagnose referred patient samples in the mobile laboratory
